# Extensive Use of Interventional Therapies Improves Survival in Unresectable or Recurrent Intrahepatic Cholangiocarcinoma

**DOI:** 10.1155/2016/8732521

**Published:** 2016-02-04

**Authors:** Ricarda Seidensticker, Max Seidensticker, Kathleen Doegen, Konrad Mohnike, Kerstin Schütte, Patrick Stübs, Erika Kettner, Maciej Pech, Holger Amthauer, Jens Ricke

**Affiliations:** ^1^Klinik für Radiologie und Nuklearmedizin, Universitätsklinikum Magdeburg, Leipziger Strasse 44, 39120 Magdeburg, Germany; ^2^Zentrum für Gastrointestinale Tumoren (ZeGIT), Universitätsklinikum Magdeburg, Leipziger Strasse 44, 39120 Magdeburg, Germany; ^3^Deutsche Akademie für Mikrotherapie (DAfMT), Leipziger Strasse 44, 39120 Magdeburg, Germany; ^4^Klinik für Gastroenterologie, Hepatologie und Infektiologie, Universitätsklinikum Magdeburg, Leipziger Strasse 44, 39120 Magdeburg, Germany; ^5^Klinik für Allgemein-, Viszeral- und Gefäßchirurgie, Universitätsklinikum Magdeburg, Leipziger Strasse 44, 39120 Magdeburg, Germany

## Abstract

*Aim*. To assess the outcomes of patients with unresectable intrahepatic cholangiocellular carcinoma (ICC) treated by a tailored therapeutic approach, combining systemic with advanced image-guided local or locoregional therapies.* Materials and Methods*. Treatment followed an algorithm established by a multidisciplinary GI-tumor team. Treatment options comprised ablation (RFA, CT-guided brachytherapy) or locoregional techniques (TACE, radioembolization, i.a. chemotherapy).* Results*. Median survival was 33.1 months from time of diagnosis and 16.0 months from first therapy. UICC stage analysis showed a median survival of 15.9 months for stage I, 9 months for IIIa, 18.4 months for IIIc, and 13 months for IV. Only the number of lesions, baseline serum CEA and serum CA19-9, and objective response (RECIST) were independently associated with survival. Extrahepatic metastases had no influence.* Conclusion*. Patients with unresectable ICC may benefit from hepatic tumor control provided by local or locoregional therapies. Future prospective study formats should focus on supplementing systemic therapy by classes of interventions (“toolbox”) rather than specific techniques, that is, local ablation leading to complete tumor destruction (such as RFA) or locoregional treatment leading to partial remission (such as radioembolization). This trial is registered with German Clinical Trials Registry (Deutsche Register Klinischer Studien), DRKS-ID: DRKS00006237.

## 1. Introduction

Peripheral or intrahepatic cholangiocellular carcinoma (ICC) is a rare neoplasm. However, its incidence and mortality have been reported to be increasing worldwide [1]. Prognosis is poor, with a 5-year survival below 5%, including patients who do undergo tumor resection. However, surgical treatment currently represents the only potentially curative therapy. Unfortunately only 20% of patients are eligible for resection because of disease spread, anatomic location, inadequate hepatic reserve, or limiting comorbidities [2–5].

Median survival for patients with untreated unresectable ICC has been reported as 3–6 months [5, 6]. Furthermore, systemic intravenous (i.v.) chemotherapy (ivCTX) has only limited benefit. Although modern chemotherapy regimens have improved survival considerably in recent years, median survival is still less than one year for, for example, gemcitabine plus cisplatin [7].

Several palliative therapeutic options exist for patients with unresectable ICC. The goals of palliative therapy are to control local tumor growth, to relieve symptoms, and to improve and preserve quality of life. Thus, local-ablative treatment options are gaining attention, as results from studies analyzing radiofrequency ablation (RFA) and ^90^Y-radioembolization (RE), high dose rate brachytherapy (HDR-BT), intra-arterial chemotherapy (iaCTX), and transarterial chemoembolization (TACE) have been encouraging [8–12].

However, most of these studies included patients with intrahepatic and extrahepatic cholangiocarcinoma or gall-bladder cancer and involved only a small number of patients, so that definitive conclusions are sometimes difficult to draw.

Since 2006, we have treated patients with unresectable or recurrent ICC by different local therapies (alone or in combination) according to a therapy algorithm that was established after thorough discussion in a multidisciplinary team (GI board) involving surgeons, gastroenterologists, medical oncologists, and interventional radiologists. Data from these patients treated according to this algorithm were prospectively collected in an institutional database.

In the study described herein we present the clinical outcomes of this patient-tailored therapeutic approach, combining systemic and image-guided local or locoregional therapies for the treatment of intrahepatic cholangiocarcinoma in nonsurgical candidates.

## 2. Materials and Methods

This study was compliant with the ethical guidelines of the 1975 Declaration of Helsinki and was approved by our Institutional Review Board (positive vote assigned by “Ethikkommission der Medizinischen Fakultät der Otto-von-Guericke-Universität Magdeburg” at 7-16-2013); written informed consent to scientific use of data was obtained before therapy. All clinical data were obtained from the prospectively maintained institutional ICC database.

The study was registered at DRKS (Deutsche Register Klinischer Studien DRKS00006237).

### 2.1. Patients

From March 2006 to June 2012 (last follow-up performed in March 2013), 75 consecutive patients with unresectable ICC were referred to our multidisciplinary GI board and received treatment recommendations with local or locoregional treatments often supplementary to systemic treatments. All of these patients were not surgical candidates due to advanced tumor stage, comorbidities, or refused resection. From this cohort, 20 patients were excluded from analysis: 10 were lost to follow-up within the first two months (most of them initially referred from distant centers) and 10 presented with a secondary malignoma (3 of those with an additional extrahepatic cholangiocellular carcinoma, i.e., Klatskin tumor). Thus, 55 patients were analyzed. Patient and tumor characteristics at the time of first local or locoregional therapy are summarized in Table 1.

**Figure 1:**
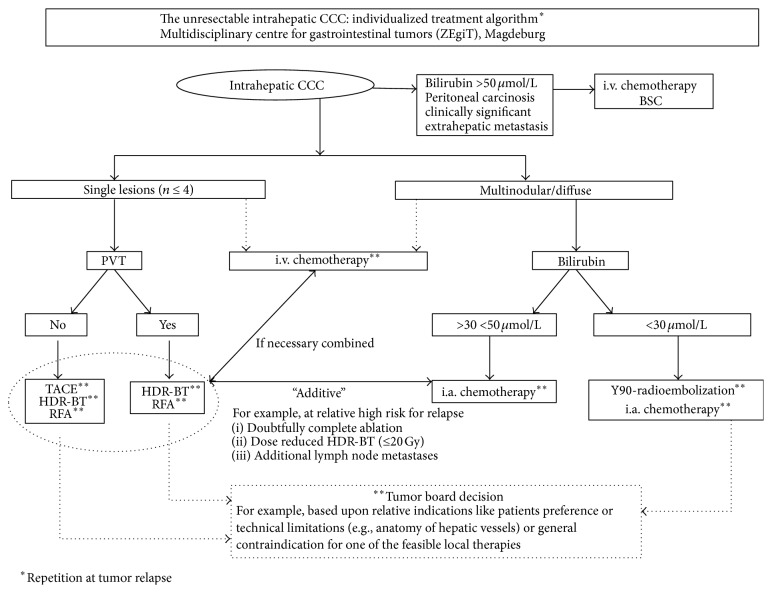
Algorithm for the treatment of intrahepatic cholangiocellular carcinoma. CCC, cholangiocellular carcinoma; HDR-BT, image-guided HDR brachytherapy; TACE, transarterial chemoembolization; RFA, radiofrequency ablation; i.a., intra-arterial; i.v., intravenous; BSC, best supportive care; PVT, portal venous thrombosis.

Palliative treatment options were part of the aforementioned multidisciplinary treatment algorithm. The algorithm is outlined in detail in Figure 1. Image-guided techniques comprised RFA (radiofrequency ablation), TACE (conventional chemoembolization), HDR-BT (CT-guided interstitial high dose rate brachytherapy), RE (^90^Y-radioembolization), iaCTX (intra-arterial chemotherapy), and ivCTX (intravenous chemotherapy).

Factors guiding the treatment allocation included stage and specific morphological properties of the disease (tumor size, number of lesions, and preexisting extrahepatic metastases) as well as liver function and performance status.

All 55 patients analyzed have been treated with at least one local or locoregional treatment option at our clinic. Patients were reassessed with clinical examination and CT or MR imaging every 3 months thereafter. According to that restaging patients were entered in the treatment algorithm again if disease progression was present. As a consequence, 37 out of 55 patients received one type of ablative or locoregional therapy, whereas another 18 patients received a combination of additional image-guided therapies. With 21 patients presenting after previous, often multiple chemotherapy lines, 16 patients received systemic treatments after the first local/locoregional intervention. All treatment details after inclusion as well as tumor-targeted prior treatments are outlined in detail in Table 1.

### 2.2. Evaluation and Staging

Diagnosis of ICC was based on biopsy. Pretreatment assessment consisted of demographics, presence or absence of cirrhosis, biliary obstruction and portal invasion, extrahepatic metastases, and prior treatments. Diagnostic imaging was performed by magnetic-resonance imaging (MRI) and/or triphasic computerized tomography (CT).

Staging was performed at the time of first diagnosis as baseline staging and again at the time of the first interventional therapy at our institution by the TNM classification adapted from the 6th edition of the staging manual of the UICC/AJCC [13]. Lymph nodes were considered to be metastatic when they were larger than 1 cm in short-axis diameter [14].

The treatment algorithm groups patients according to six potential treatments (Figure 1). Patients with single tumors (*n* ≤ 4) received HDR-BT, TACE, or RFA in the absence of portal vein thrombosis (PVT). In case of PVT, only HDR-BT or RFA were applicable. Concomitant chemotherapy was recommended in patients with biologically aggressive tumors (disease free interval < 12 months) specifically in chemotherapy-naïve patients. In patients with biologically favorable tumors with disease free interval ≥ 12 months, an ECOG > 1, and/or previous chemotherapies further chemotherapies immediately after complete ablative or locoregional treatment were not recommended.

Patients with multinodular (*n* > 4) or diffuse disease received radioembolization or iaCTX with 5-fluorouracil/leucovorin (5-FU/LV) when bilirubin was less than 30 *μ*mol/L. If bilirubin was 30–50 *μ*mol/L, iaCTX was preferred alone or in combination with HDR-BT or RFA (depending on the likelihood for reliable, technically safe complete tumor destruction). Patients with bilirubin above 50 *μ*mol/L and those with diffuse peritoneal carcinomatosis were not eligible for any local-ablative or locoregional therapy and received ivCTX or best supportive care only. All treatment recommendations were issued by the multidisciplinary gastrointestinal oncology team in consensus.

Complications were classified following CTCAE v4.0, with minor (CTCAE Grades 1 and 2) or major complications (CTCAE Grades 3 and 4).

### 2.3. Local-Ablative Therapies

#### 2.3.1. Radiofrequency Ablation (RFA)

RFA is an ablative technique intending complete local tumor destruction. RFA was performed under CT or MRI guidance using a radiofrequency applicator, which can be expanded stepwise to cover an area of maximum diameter 5 cm and a 150 W RF generator (Starburst Semi-Flex; AngioDynamics, Mountain View, CA) [15, 16]. The RFA protocol was always completed according to the manufacturer's instructions; completeness of ablation was confirmed by MRI 24 hours after RFA.

#### 2.3.2. Image-Guided HDR Brachytherapy (HDR-BT)

The technique of HDR-BT has been described in detail elsewhere [9, 17]. As an ablative technique, its intention is complete and durable local tumor destruction. In brief, liver malignancies are treated with high dose rates of iridium-192 in an afterloading technique after percutaneous positioning of the brachytherapy catheters under CT or MRI control. The prescribed minimum dose for the clinical target volume is 20 Gy. Specifically in patients where RFA is not feasible owing to larger tumor sizes (>5 cm) or adjacent, potentially cooling structures such as larger vessels, HDR-BT is a useful option [18–20].

#### 2.3.3. Radioembolization (RE) with Yttrium-90 Microspheres

Radioembolization with ^90^Y-labeled resin microspheres has been shown to be effective in unresectable ICC and tumor metastases of the liver [21, 22]. Its intended effect was partial remission of diffuse hepatic tumor spread rather than complete tumor ablation.

The principle of RE is based on the dual blood supply of the liver from the portal vein and the hepatic artery, so delivery of the radioactive microspheres via the hepatic artery results in high dose local irradiation with only minor effects on normal liver tissue. All patients underwent pretreatment mesenteric angiography and ^99^Tc-macroaggregated albumin scanning to minimize the risk of nontarget embolisation [19, 23]. A detailed account of the treatment protocol has been published previously [22]. The median dose was 1.63 GBq (range 0.9–2.55 GBq).

#### 2.3.4. Transarterial Chemoembolization (TACE)

The intended effect of TACE was partial remission of limited hepatic tumor spread beyond the technical capabilities of local ablation such as through RFA or CT-guided brachytherapy. TACE was conducted by standard techniques with an emulsion of doxorubicin and cisplatin in lipiodol (1 mL contains 0.5 mL lipiodol and 2.5 mg each of doxorubicin and cisplatin) until stasis in tumor feeding arteries was achieved. No additional embolization particles were administered. TACE was performed every 6 weeks. After three sessions tumor response was assessed by CT and/or MRI and, depending on outcome, TACE was either continued or interrupted [19, 24, 25].

#### 2.3.5. Intra-Arterial Chemotherapy (iaCTX)

iaCTX was performed on an outpatient basis. Chemotherapy was delivered through a microcatheter-port system into the hepatic artery, implanted via the common femoral artery as described elsewhere [26]. This method potentially decreases systemic side effects (e.g., nausea and vomiting) and may optimize the chemotoxic effects of the drugs in the hepatic malignancy [27, 28].

Intra-arterial chemotherapy consisted of daily infusions of fluorouracil (5-FU) 600 mg/m² and folinic acid 170 mg/m² on days 1–5, repeated on day 22.

Nine patients (16%) received a median of 6 cycles (range 4–23 cycles) of intra-arterial 5-FU chemotherapy.

#### 2.3.6. Intravenous Chemotherapy (ivCTX)

Lacking a well-defined therapeutic standard until 2010, various ivCTX regimens have been administered following protocols including monotherapy or combinations of cisplatin, gemcitabine, oxaliplatin, 5-FU/FA, and capecitabine [7, 29, 30]. Since 2010 the standard first-line therapy was gemcitabine combined with cisplatin [7]. In our study, sixteen patients received ivCTX in combination with their local therapy. The median number of chemotherapeutic cycles was 5 (range 1–12). Thirteen patients (24%) received one line of ivCTX, two (4%) received a second line, and one (2%) received a third line. Patients who had been treated with ivCTX only are not part of this analysis.

### 2.4. Follow-Up/Clinical Assessments

At imaging follow-up, usually every three months after the intervention, clinical assessment and laboratory tests (blood counts, liver function tests, and tumor markers (carbohydrate antigen 19-9 (CA 19-9) and carcinoembryonic antigen (CEA))) were routinely performed.

Patients diagnosed with progressing ICC during follow-up were reassessed by the multidisciplinary treatment decision algorithm and treated again accordingly. Patients were followed until death or censored at the last known clinical follow-up.

### 2.5. Imaging Analysis

Patients were examined every three months by liver MRI using the liver-specific contrast agent gadoxetic acid (Gd-EOB-DTPA, Primovist, Bayer Healthcare, Berlin, Germany) or triphasic contrast-enhanced CT with iopremol 300 (Imeron 300, Bracco Imaging, SpA, Milan, Italy) of the abdomen. Every six months a chest X-ray was conducted and once a year a multislice CT of the thorax. Response was assessed applying the RECIST 1.1 criteria [31].

### 2.6. Statistical Analysis

Descriptive statistics were calculated for quantitative variables; frequency counts by category were calculated for qualitative variables; 95% confidence intervals are presented where appropriate.* p* values were considered significant if <0.05. The primary study endpoint was overall survival (estimated from the date of first interventional therapy at our institution and additionally from the date of first diagnosis), analyzed by the Kaplan-Meier method and compared between different groups by a log-rank test.

The following prognostic factors for influencing patient survival were evaluated: patient's age and sex, time interval from first diagnosis to first local therapy at our institution, performance status at the time of first local therapy at our institution (Karnofsky and ECOG), prior resection, prior chemotherapies, prior local therapies, tumor load, tumor number, tumor size and tumor stage (according to UICC), extrahepatic metastasis, vascular infiltration, portal vein invasion, biliary obstruction, ascites, cirrhosis, elevated tumor marker levels (CEA and CA 19-9), best response, and therapy-associated complications.

Several prognostic factors were grouped for analysis of differences in survival. These are listed below (Table 3).

Univariate and multivariate Cox regression analyses were performed to identify factors associated with the patients' survival. Only factors showing significance (*p* < 0.05) in the univariate model were included in the multivariate analysis.

Statistical analyses were performed with SPSS (version 21, IBM, Chicago, IL, USA).

## 3. Results

### 3.1. Patient Population

Table 1 summarizes the patient and tumor characteristics in the current study.

At the time of first interventional treatment, 58% of the patients suffered from bilobar tumor spread, the median tumor size was 45 mm, and 65% presented with extrahepatic metastasis (lymph node metastasis (*n* = 32), single peritoneal nodules (*n* = 8), and pulmonary (*n* = 5) and bone metastasis (*n* = 2)).

Forty-two patients (76%) underwent prior therapies before local intervention at our institution, 21 (38%) had undergone liver-directed therapy, and another 21 patients (38%) had received ivCTX.

### 3.2. Treatment Characteristics and Complication Rates

Treatment characteristics and Grade 3-4 treatment-related toxicities of all 55 patients are summarized in Table 2.

For 101 sessions of HDR brachytherapy, 3 (3%) Grade 3 events (no Grade 4) were reported. Of 16 patients who received ivCTX combined with a local therapy, 9 (56%) suffered from Grade 3 toxicities (no Grade 4). Patients receiving iaCTX, TACE, RE, or RFA did not report any Grade 3 or 4 toxicity. No patient suffered from severe liver decompensation.

### 3.3. Best Tumor Response

Of 55 patients, 8 (15%) showed complete remission, 21 (38%) partial remission, 8 (15%) stable disease, and 18 (33%) progressive disease. The best response for each type of therapy is shown along with the treatments in Table 2.

### 3.4. Follow-Up and Overall Survival

**Figure 2:**
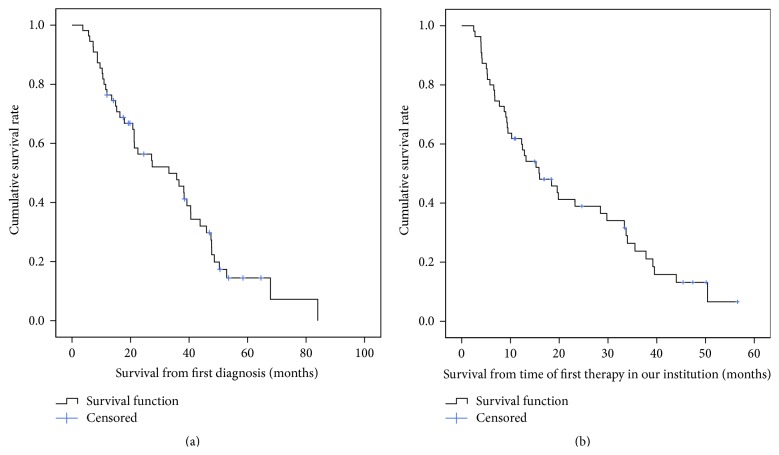
Overall survival rate in all patients from time of first diagnosis (a) and from time of first therapy at our institution (b). Median overall survival from time of first diagnosis: 33.1 months (95% CI 16.5–49.8 months). Median overall survival from time of first therapy at our institution: 16.0 months (95% CI 8.8–32.2 months).

Median follow-up time was 11.7 months (range 0.9–51.1). Forty-three of the 55 (78.2%) patients died during the follow-up period. The median number of follow-up visits was 3 (range: 1–15). The median overall survival period was 33.1 months (95% CI 16.5–49.8 months) from the time of first diagnosis and 16.0 months (95% CI 8.8–32.2 months) from the time of first local therapy at our institution (Figures 2(a) and 2(b)). A subgroup analysis by UICC stage showed a median survival of 15.9 months (95% CI 11.9–19.9 months) for patients with stage I disease, 9 months (95% CI 0.8–17.2 months) for patients with stage IIIa, 18.4 months (95% CI 8.1–28.7 months) for patients with stage IIIc, and 13 months (95% CI 6–18.9 months) for patients with stage IV. Only 5 patients were in stage II when they received first local therapy and, of these, 3 were still alive and therefore censored at the time of analysis. There was no significant difference in survival between the various stages.

### 3.5. Factors Related to Patients' Survival Period

**Figure 3:**
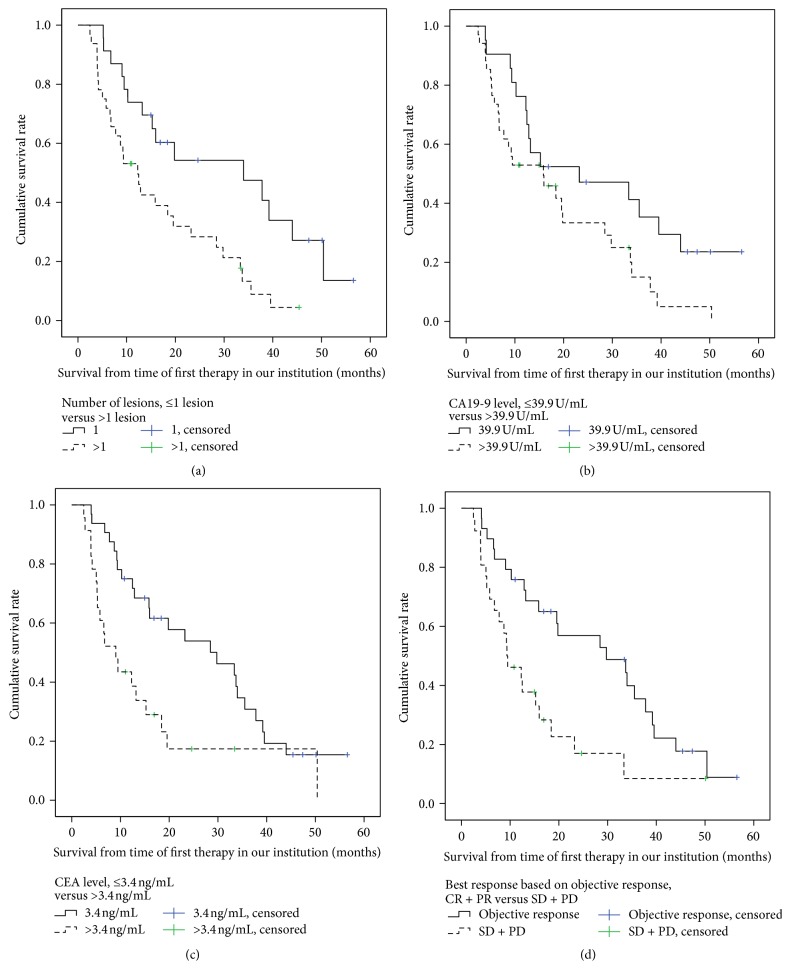
Overall survival rate in all patients according to influencing factors (derived from Cox model, Table 3). (a) Overall survival rates with regard to number of lesions (≤1 lesion versus >1 lesion), with a median overall survival of 34 and 12.3 months, *p* = 0.006. (b) Overall survival rates with regard to CA 19-9 level (≤39.9 U/mL versus >39.9 U/mL) with a median overall survival of 23.2 and 15.9 months, *p* = 0.043. (c) Overall survival rates with regard to CEA level (≤3.4 ng/mL versus >3.4 ng/mL), with a median overall survival of 29.8 and 9.1 months, *p* = 0.007. (d) Overall survival rates with regard to best response (objective response (CR and PR) versus stable and progressive disease (SD + PD)), with a median overall survival of 29.8 and 9.3 months, *p* = 0.005.

The following variables were found to be significant in the univariate analysis (Table 3) and were entered into the multivariate Cox regression model: number of tumor lesions, the tumor markers carcinoembryonic antigen (CEA) and carbohydrate antigen 19-9 (CA 19-9), and objective response. The multivariate analysis showed that these parameters were independent factors associated with duration of survival after therapy. According to the Kaplan-Meier analysis, factors identified as influencing median overall survival (after first local treatment) were number of tumors (1 versus ≥2), 34 versus 12.3 months, *p* = 0.006; elevated CA 19-9 levels (normal versus above normal), 23.2 versus 15.9 months, *p* = 0.043; elevated CEA levels (normal versus above normal), 29.8 versus 9.1 months, *p* = 0.007 (the upper normal limits were taken to be 39.9 U/mL for CA 19-9 and 3.4 ng/mL for CEA); and objective response according to RECIST, 29.8 versus 9.3 months, *p* = 0.005. Corresponding survival curves are shown in Figures 3(a)–3(d).

## 4. Discussion

ICC (intrahepatic cholangiocarcinoma of the mass-forming type) is a uniformly fatal disease with a poor prognosis when detected at an advanced stage. Unfortunately most patients present with unresectable disease because of the absence of symptoms until late in disease progression. Published data concerning systemic or local therapy options are limited. Furthermore, most studies fail to provide a clear profile of their patients in respect of tumor stage or metastatic disease and often comprise heterogeneous study populations including patients with Klatskin tumors, ampullary carcinoma, and gallbladder carcinoma. Therefore, direct comparison with systemic or standard locoregional therapy approaches is sometimes difficult.

We sought to investigate the outcome of a patient-tailored therapy course, including all modalities of minimally invasive oncology, applied alone or in combination, singly or repeatedly, following an interdisciplinary treatment algorithm for patients with mass-forming ICC only. In our study the clinical stage of patients was well described, and tumor disease was staged according to the UICC tumor node metastasis (TNM) classification system.

Our study showed a median survival of 16 months from first local therapy and 33.1 months from first diagnosis, which is higher than that found in most of the earlier studies examining different locoregional therapies. Kiefer et al. [12] reported a median survival of 15 months from chemoembolization and 20 months from diagnosis. In their study 62 patients with heterogeneous tumor entities were treated, 37 with histologically proven ICC and 25 with poorly differentiated adenocarcinoma of unknown primary origin; 49% of the patients presented with UICC (TNM) stage IIIa and 24% with stage IV, comparable to our study where 40% of patients presented with stage IIIc and 25% with stage IV. Survival data concerning different UICC stages are unfortunately not reported.

In a study conducted by Park et al. 72 patients (61% stage IIIa/IIIb and 19% stage IV) with untreated, unresectable ICC received TACE as first-line therapy. Survival after diagnosis was measured and compared with that of patients who received supportive therapy only [10]. Median survival was shorter than in our study: 12.2 months for the TACE group and 3.3 months for the “supportive treatment” group.

Another study assessing survival after RE was published by an Australian group in 2010. In that study, 25 patients underwent RE in advanced ICC: 60% had >25% tumor burden, 48% showed extrahepatic metastasis, and 76% had previous antitumor treatments. Seven patients (26%) underwent ivCTX after RE. The median survival after diagnosis of ICC was 20.4 months and after RE 9.3 months, but for 13 patients with hepatic disease only a median survival of 16.3 months was achieved [8].

Excellent results have been reported for 13 patients with 17 unresectable but small ICC (10 tumors < 3 cm, 5 tumors 3–5 cm, and 2 tumors > 5 cm) treated by RFA in an early tumor stage (8 stage I, 3 stage II, 1 stage IIIb, and 1 stage IV). They presented a median overall survival period of 38.5 months [11].

Schnapauff et al. evaluated outcomes after repeated interstitial HDR-BT (27 sessions) in 15 patients with unresectable ICC who did not show extrahepatic metastasis and suffered from limited hepatic disease only (<5 lesions), revealing a median survival of 11 months and 21 months after primary diagnosis [9].

Recently, results of a larger-scale randomized phase III trial of systemic therapy were published, comparing “gemcitabine alone” with “gemcitabine plus cisplatin” in a heterogeneous group of 410 patients with locally advanced or metastatic cholangiocarcinoma, gall-bladder cancer, or ampullary cancer. In that study the gemcitabine-cisplatin combination resulted in a significantly prolonged median overall survival of 11.7 months, compared with 8.1 months in the gemcitabine monotherapy group [7].

In summary, comparing our results with those from other studies on local-ablative therapies on ICC, we can conclude a comparatively long overall survival of our patient cohort, even though the stage of disease was mostly advanced according to UICC. Overall survival in our study cohort was substantially longer than in recent ivCTX-only studies [7]. However, since a significant proportion of our patients were already heavily pretreated with various treatments (including ivCTX) when receiving a first locoregional treatment of the liver, a selection bias towards a favorable tumor biology cannot be ruled out. However, irrespective of this potential bias, we were able to show an overall survival from first diagnosis that was comparable to that after surgical resection with curative intent (median survival of 27–36 months) [32–34].

In the present study 65% (36/55) of the patients had extrahepatic metastases (Table 1) before first treatment at our institution. In agreement with Gusani et al. [35] who reported the treatment outcome of ICC after TACE, we found that median survival after therapy did not differ significantly between patients with liver-only disease and patients suffering from extrahepatic metastasis as well. Similar findings have emerged from other studies on TACE and radioembolization of ICC [8, 12]. Additionally, overall survival was not affected by the UICC stage at the time of treatment at our institution. Regarding tumor characteristics, only the number of ICC lesions had an influence on survival (1 versus >1 lesion, *p* = 0.006). We claim that all these results indicate a pivotal change in the management and treatment of patients with advanced ICC disease. The importance of local tumor control as the main palliative goal has to be emphasized, regardless of extrahepatic metastases and stage of disease. This assumption is underlined by the finding that objective tumor response (liver only) was one of the independent factors influencing survival, with 29.8 months for OR and 9.3 months for SD/PD (*p* = 0.005). Obviously, prevention of liver failure due to progression of intrahepatic tumor (a frequent cause of mortality) is of utmost importance. According to our and others' results, effective suppression of liver tumors may prolong the survival period even in patients with advanced local disease and extrahepatic metastasis. We strongly believe that these findings should further promote clinical trials of local or locoregional therapies and that these may become a key modality in the treatment of nonresectable ICC in future.

Besides objective response and the number of ICC manifestations, only elevation of serum tumor markers CA 19-9 and CEA beyond normal levels showed a negative influence on survival. This might represent a more active tumor biology in patients with elevated tumor markers. Other factors included in our analysis (patient age and gender, prior liver-directed therapies, tumor size and stage, unilobar or bilobar tumor spread, portal vein thrombosis, vascular invasion, biliary obstruction, ascites, cirrhosis, therapy-related complications, ECOG status, Karnofsky index, and time from primary diagnosis to first local therapy) did not appear to affect outcome.

## 5. Conclusion

Our results show that patients with unresectable ICC of the mass-forming type may benefit from hepatic tumor control by local or locoregional therapies even with presence of extrahepatic spread. If local or locoregional therapies were deemed favorable by clinical means, therapeutic recommendations for a specific technique were driven by technical strengths or limitations of a given modality. As such, future prospective study formats should focus on supplementing systemic therapy by classes of interventions (“toolbox”) rather than specific techniques, that is, local ablation leading to complete tumor destruction (such as RFA) or locoregional treatment leading to partial remission (such as radioembolization or TACE).

## Figures and Tables

**Table 1:** Patients' characteristics.

Demographics and disease history		%/(range)
Total *N*	55	100%
Sex		
Male	28	50.9%
Female	27	49.1%
Age, year		
Median	67.3	(34.0–82.6)
≤65	25	45.5%
>65	30	54.5%
Months from diagnosis to 1st therapy		
Median	10	(0.8–64.4)
Karnofsky index, *n* = 47		
Median	70	(60–100)
60	9	16.4%
70	13	23.4%
80	14	25.6%
90	17	31.0%
100	2	3.6%
ECOG index, *n* = 47		
Median	1	(0–2)
0	19	34.6%
1	27	49.0%
2	9	16.4%
Prior liver-directed treatment (*n*)		
Any	21	38.2%
Resection	15	27.3%
Intraoperative RFA	3	5.5%
TACE	2	3.6%
RFA	1	1.8%
Prior chemotherapy (*n*)		
Yes	21	38.2%
No	34	61.8%
Prior chemotherapy lines (*n*)		
One	17	30.9%
Two	2	3.6%
>two	2	3.6%
Median	1	(1–5)
Prior chemotherapy agents (*n*)		
Gemcitabine	19	34.5%
Oxaliplatin	12	21.8%
Capecitabine	8	14.5%
5-FU/FA	4	7.3%
Cisplatin	3	5.5%
Others^*∗*^	9	16.4%
T-stage (*n*)		
T1	21	38.2%
T2	12	21.8%
T3	21	38.2%
T4	1	1.8%
Overall tumor stage (UICC^*∗∗*^) (*n*) at first diagnosis		
Stage I	17	30.9%
Stage II	3	5.5%
Stage IIIa	3	5.5%
Stage IIIb	0	0%
Stage IIIc	21	38.2%
Stage IV	5	9.1%
No information available	6	10.9%
Overall tumor stage (UICC^*∗∗*^) (*n*) at first local therapy in Magdeburg		
Stage I	11	20.0%
Stage II	5	9.1%
Stage IIIa	3	5.5%
Stage IIIb	0	0%
Stage IIIc	22	40.0%
Stage IV	14	25.5%
CEA		
Median, range [ng/mL]	2.6	(0.3–391.7)
Elevated, >3.4 ng/mL (*n*)	23	41.8%
Not elevated (*n*)	32	58.2%
CA 19–9		
Median, range [U/mL]	66	(0.6–72.9)
Elevated, >39.9 U/mL (*n*)	34	61.8%
Not elevated (*n*)	21	38.2%
Tumor load		
Median, range (%)	8	(2–80)
Tumor size		
Median, range (mm)	45	(14–189)
Extent of disease (*n*)		
Bilobar	32	58.2%
Unilobar	23	41.8%
Extrahepatic metastases (*n*)		
All	36	65.5%
Lymph node metastases	32	58.2%
Peritoneal metastases	8	14.5%
Pulmonary metastases	5	9.1%
Bone metastases	2	3.6%
Concomitant liver disease (*n*)		
Vascular infiltration	21	38.2%
Cirrhosis	20	36.4%
Biliary obstruction	18	32.7%
Portal vein thrombosis	10	18.2%
Ascites	7	12.7%
Therapies and combinations of therapies (*n*)		
HDR-BT	19	34.5%
RE	5	9.1%
TACE	2	3.6%
RFA	1	1.8%
HDR-BT & ivCTX	11	20.0%
HDR-BT & iaCTX	6	10.9%
HDR-BT & RE	3	5.5%
HDR-BT & RFA	2	3.6%
HDR-BT & iaCTX & ivCTX	2	3.6%
HDR-BT & RE & ivCTX	2	3.6%
TACE & ivCTX	1	1.8%
RE & iaCTX	1	1.8%

^*∗*^Irinotecan (*n* = 1), taxotere (*n* = 1), bevacizumab (*n* = 1), erlotinib (*n* = 1), mitomycin C (*n* = 1), cetuximab (*n* = 2), and sorafenib (*n* = 2).

^*∗∗*^Acc. to UICC Edition 6, stage I disease is a solitary tumor without vascular involvement; stage II disease is a solitary tumor with vascular invasion or multiple tumors <5 cm; stage IIIa disease is multiple tumors >5 cm with or without vascular invasion; stage IIIb disease is perforation of the peritoneum or infiltration of adjacent organs; stage IIIc disease is any tumor with regional lymph node metastasis; and stage IV disease is any tumor with distant metastasis.

**Table 2:** Treatment characteristics and cumulative toxicities analysis: only Grade 3-4 toxicities are reported (CTCAE version 4.0).

Treatment characteristics	HDR-BT	RE	RFA	TACE	ivCTX	iaCTX
Patients (*n*)	45	11	3	3	16	9
Karnofsky index, median (range)	80 (60–100)	80 (60–90)	90 (60–90)	70 (70)	70 (60–100)	90 (80–90)
ECOG index, median (range)	1 (0–2)	1 (0–2)	0 (0–2)	1 (1-1)	1 (0–2)	0 (0-1)
Number of days hospitalized, median (range)	4 (1–11)	4 (3–5)	5 (4–6)	4 (3–6)	0	0
Total number of treatments/chemotherapeutic cycles (*n*)	101	20	3	12	64	43
Median number of treatments/chemotherapeutic cycles per patient (range)	1 (1–5)	1 (1–4)	3 (1-1)	4 (3–5)	5 (1–12)	6 (4–23)
Median RE-dose delivered (GBq), median (range)	—	1.63 (0.9–2.55)	—	—	—	—
Best response	CR	PR	CR	PD	SD	PR
Adverse events acc. CTCAE (highest grade recorded)	3	2	2	1	3	2
Abscess (*n*)	1	—	—	—	—	—
Shivering^*∗*^ (*n*)	1	—	—	—	—	—
Hematoma subcapsular (*n*)	1	—	—	—	—	—
Anemia (*n*)	—	—	—	—	1	—
Thrombopenia (*n*)	—	—	—	—	1	—
Neutropenia (*n*)	—	—	—	—	1	—
Anorexia (*n*)	—	—	—	—	1	—
Fatigue (*n*)	—	—	—	—	2	—
Pain (*n*)	—	—	—	—	1	—
Diarrhea (*n*)	—	—	—	—	1	—
Rash (*n*)	—	—	—	—	1	—

Data are expressed as absolute number of events (*n*).

CR, complete response; PR, partial response; SD, stable disease; PD, progressive disease. CTCAE, common toxicity criteria of adverse events.

^*∗*^Shivering due to radiation effects during HDR-BT with the need for abruption of the intervention.

**Table 3:** Cox regression analysis of the prognostic factors of the patient survival period.

Variables	Univariate analysis			Multivariate analysis		
	HR	95% CI	*p*	HR	95% CI	*p*
Age (≤65 years versus >65 years)	0.83	0.45–1.53	0.551			
Sex (male versus female)	1.17	0.64–2.12	0.615			
Previous resection (no versus yes)	0.73	0.37–1.43	0.358			
UICC at first therapy (stage I versus stages II–IV)	1.20	0.96–1.50	0.133			
Lobar spread of disease (unilobar versus bilobar)	1.47	0.78–2.76	0.237			
Extrahepatic metastasis (no versus yes)	1.55	0.78–3.09	0.211			
Tumor load (≤10% versus >10%)	1.81	0.99–3.31	0.055			
Number of lesions (1 versus >1)	2.44	1.27–4.71	**0.008**	2.85	1.43–5.65	**0.003**
Portal vein thrombosis (no versus yes)	1.43	0.62–3.30	0.407			
Vascular infiltration (no versus yes)	1.20	0.65–2.24	0.560			
Ascites (no versus yes)	1.49	0.66–3.35	0.314			
Liver cirrhosis (no versus yes)	1.25	0.67–2.34	0.493			
Biliary obstruction (no versus yes)	1.02	0.53–1.97	0.950			
ECOG index (0 versus 1–4)	1.23	0.66–2.30	0.511			
CA19–9 (≤39.9 U/mL versus >39.9 U/mL)	1.93	1.01–3.68	**0.047**	2.05	0.99 – 4.22	**0.052**
CEA (≤3.4 ng/mL versus >3.4 ng/mL)	2.30	1.23–4.31	**0.009**	1.89	0.97 – 3.72	**0.025**
Objective response (CR + PR versus SD + PD)	2.43	1.28–4.60	**0.006**	2.84	1.41 – 5.72	**0.003**
Complications (no versus yes)	1.06	0.68–1.67	0.796			
Tumor size (≤50 mm versus >50 mm)	1.35	0.74–2.46	0.328			
Tumor size (≤100 mm versus >100 mm)	1.22	0.60–2.50	0.585			

HR, hazard ratio; CI, confidence interval; objective response categories, see Table 2.

## Abbreviations

### Abbreviations

ICC: Intrahepatic cholangiocellular carcinoma
CTCAE: National Cancer Institute Common Toxicity Criteria
95% CI: 95% confidence interval
CEA: Carcinoembryonic antigen
CA 19-9: Carbohydrate antigen 19-9
RECIST: Response evaluation criteria in solid tumors
i.v.: Intravenous
IvCTX: Intravenous chemotherapy
RFA: Radiofrequency ablation
RE: ^90^Y-radioembolization
HDR-BT: High dose rate brachytherapy
IaCTX: Intra-arterial chemotherapy
TACE: Transarterial chemoembolization
UICC: Union for International Cancer Control
ECOG: Eastern Cooperative Oncology Group
MRI: Magnetic-resonance imaging
CT: Computerized tomography
PVT: Portal vein thrombosis
5-FU/FA: 5-Fluorouracil/leucovorin
TNM: Tumor node metastasis.

## References

[1] Blechacz B., Gores G. J. (2008). Cholangiocarcinoma: advances in pathogenesis, diagnosis, and treatment. *Hepatology*.

[2] Yamamoto J., Kosuge T., Takayama T. (1992). Surgical treatment of intrahepatic cholangiocarcinoma: four patients surviving more than five years. *Surgery*.

[3] Lieser M. J., Barry M. K., Rowland C., Ilstrup D. M., Nagorney D. M. (1998). Surgical management of intrahepatic cholangiocarcinoma: a 31-year experience. *Journal of Hepato-Biliary-Pancreatic Surgery*.

[4] Chu K. M., Lai E. C., Al-Hadeedi S. (1997). Intrahepatic cholangiocarcinoma. *World Journal of Surgery*.

[5] Khan S. A., Thomas H. C., Davidson B. R., Taylor-Robinson S. D. (2005). Cholangiocarcinoma. *The Lancet*.

[6] Cunningham S. C., Choti M. A., Bellavance E. C., Pawlik T. M. (2007). Palliation of hepatic tumors. *Surgical Oncology*.

[7] Valle J., Wasan H., Palmer D. H. (2010). Cisplatin plus gemcitabine versus gemcitabine for biliary tract cancer. *The New England Journal of Medicine*.

[8] Saxena A., Bester L., Chua T. C., Chu F. C., Morris D. L. (2010). Yttrium-90 radiotherapy for unresectable intrahepatic cholangiocarcinoma: a preliminary assessment of this novel treatment option. *Annals of Surgical Oncology*.

[9] Schnapauff D., Denecke T., Grieser C. (2012). Computed tomography-guided interstitial HDR brachytherapy (CT-HDRBT) of the liver in patients with irresectable intrahepatic cholangiocarcinoma. *CardioVascular and Interventional Radiology*.

[10] Park S.-Y., Kim J. H., Yoon H.-J., Lee I.-S., Yoon H.-K., Kim K.-P. (2011). Transarterial chemoembolization versus supportive therapy in the palliative treatment of unresectable intrahepatic cholangiocarcinoma. *Clinical Radiology*.

[11] Kim J. H., Won H. J., Shin Y. M., Kim K.-A., Kim P. N. (2011). Radiofrequency ablation for the treatment of primary intrahepatic cholangiocarcinoma. *American Journal of Roentgenology*.

[12] Kiefer M. V., Albert M., McNally M. (2011). Chemoembolization of intrahepatic cholangiocarcinoma with cisplatinum, doxorubicin, mitomycin C, ethiodol, and polyvinyl alcohol. *Cancer*.

[13] Sobin L., Gospodarowicz M. K., Wittekind C. (2002). *TNM Classification of Malignant Tumours*.

[14] Yoon Y. C., Lee K. S., Shim Y. M., Kim B.-T., Kim K., Kim T. S. (2003). Metastasis to regional lymph nodes in patients with esophageal squamous cell carcinoma: CT versus FDG PET for presurgical detection prospective study. *Radiology*.

[15] Park S.-Y., Kim J. H., Won H. J., Shin Y. M., Kim P. N. (2011). Radiofrequency ablation of hepatic metastases after curative resection of extrahepatic cholangiocarcinoma. *American Journal of Roentgenology*.

[16] Carrafiello G., Laganà D., Cotta E. (2010). Radiofrequency ablation of intrahepatic cholangiocarcinoma: preliminary experience. *CardioVascular and Interventional Radiology*.

[17] Ricke J., Wust P., Stohlmann A. (2004). CT-guided interstitial brachytherapy of liver malignancies alone or in combination with thermal ablation: phase I-II results of a novel technique. *International Journal of Radiation Oncology Biology Physics*.

[18] Rühl R., Ricke J. (2006). Image-guided micro-therapy for tumor ablation: from thermal coagulation to advanced irradiation techniques. *Onkologie*.

[19] Mahnken A. H., Bruners P., Günther R. W. (2008). Techniques of interventional tumor therapy. *Deutsches Arzteblatt*.

[20] Wieners G., Pech M., Hildebrandt B. (2009). Phase II feasibility study on the combination of two different regional treatment approaches in patients with colorectal ‘liver-only’ metastases: hepatic interstitial brachytherapy plus regional chemotherapy. *CardioVascular and Interventional Radiology*.

[21] Hoffmann R.-T., Paprottka P. M., Schön A. (2012). Transarterial hepatic yttrium-90 radioembolization in patients with unresectable intrahepatic cholangiocarcinoma: factors associated with prolonged survival. *CardioVascular and Interventional Radiology*.

[22] Seidensticker R., Denecke T., Kraus P. (2012). Matched-pair comparison of radioembolization plus best supportive care versus best supportive care alone for chemotherapy refractory liver-dominant colorectal metastases. *CardioVascular and Interventional Radiology*.

[23] Kucuk O. N., Soydal C., Lacin S., Ozkan E., Bilgic S. (2011). Selective intraarterial radionuclide therapy with Yttrium-90 (Y-90) microspheres for unresectable primary and metastatic liver tumors. *World Journal of Surgical Oncology*.

[24] Kim J. H., Yoon H.-K., Sung K.-B. (2008). Transcatheter arterial chemoembolization or chemoinfusion for unresectable intrahepatic cholangiocarcinoma: clinical efficacy and factors influencing outcomes. *Cancer*.

[25] Vogl T. J., Zangos S., Eichler K. (2012). Radiological diagnosis and intervention of cholangiocarcinomas (CC). *Fortschr Röntgenstr*.

[26] Ricke J., Hildebrandt B., Miersch A. (2004). Hepatic arterial port systems for treatment of liver metastases: factors affecting patency and adverse events. *Journal of Vascular and Interventional Radiology*.

[27] Cantore M., Mambrini A., Fiorentini G. (2005). Phase II study of hepatic intraarterial epirubicin and cisplatin, with systemic 5-fluorouracil in patients with unresectable biliary tract tumors. *Cancer*.

[28] Vogl T. J., Zangos S., Eichler K., Selby J. B., Bauer R. W. (2008). Palliative hepatic intraarterial chemotherapy (HIC) using a novel combination of gemcitabine and mitomycin C: results in hepatic metastases. *European Radiology*.

[29] Skipworth J. R. A., Olde Damink S. W. M., Imber C., Bridgewater J., Pereira S. P., Malagó M. (2011). Review article: surgical, neo-adjuvant and adjuvant management strategies in biliary tract cancer. *Alimentary Pharmacology and Therapeutics*.

[30] Fuchs M., Schepp W. (2006). Is there a nonsurgical therapeutic approach to cholangiocellular carcinomas?. *Chirurg*.

[31] Eisenhauer E. A., Therasse P., Bogaerts J. (2009). New response evaluation criteria in solid tumours: revised RECIST guideline (version 1.1). *European Journal of Cancer*.

[32] Endo I., Gonen M., Yopp A. C. (2008). Intrahepatic cholangiocarcinoma: rising frequency, improved survival, and determinants of outcome after resection. *Annals of Surgery*.

[33] Yedibela S., Demir R., Zhang W., Meyer T., Hohenberger W., Schönleben F. (2009). Surgical treatment of mass-forming intrahepatic cholangiocarcinoma: an 11-year western single-center experience in 107 patients. *Annals of Surgical Oncology*.

[34] Dhanasekaran R., Hemming A. W., Zendejas I. (2013). Treatment outcomes and prognostic factors of intrahepatic cholangiocarcinoma. *Oncology Reports*.

[35] Gusani N. J., Balaa F. K., Steel J. L. (2008). Treatment of unresectable cholangiocarcinoma with gemcitabine-based transcatheter arterial chemoembolization (TACE): a single-institution experience. *Journal of Gastrointestinal Surgery*.

